# Supplementary material for: FastTrack: An open-source software for tracking varying numbers of deformable objects
Source: PLoS Comput Biol. 2021 Feb 11;17(2):e1008697. doi: 10.1371/journal.pcbi.1008697 (PMC7904165; doi:10.1371/journal.pcbi.1008697)
Supplement: S1 Table — Description and credentials of the data that have been used for testing the FastTrack software. All movies in the dataset can be downloaded at http://data.ljp.upmc.fr/datasets/TD2. (PDF) [file pcbi.1008697.s001.pdf]

S1 Table. Two-Dimensional Tracking Dataset.

Description and credentials of the data that have been used for testing the FastTrack software. All movies in the dataset can be downloaded at http://data.ljp.upmc.fr/datasets/TD2.

Identifier	Color	Number of images	Image size	Number of objects	Object type	Short description	Authors	Reference	Overlaps	Preprocess
ACT_001	No	1,001	2040x2040	1141	Active particles	Vibrated polar monodisperse discs in a liquid-like configuration.	Olivier Dauchot	17	No	No
ACT_002	No	1,001	1121x996	1085	Active particles	Vibrated polar monodisperse discs in a cristalline-like configuration.	Olivier Dauchot	18	No	Yes
ACT_003	No	1,000	1728x1728	853	Active liquid	Vibrated polar monodisperse discs.	Olivier Dauchot	19	No	Yes
ACT_004	No	998	4096x3072	*Variable*	Active droplets	Motion of water droplets in an oil-surfactant medium of squalane and monoolein.	Olivier Dauchot	20	No	No
ART_001	No	2,000	1280x1024	*Variable*	Brine schrimps	Brine shrimps (*Artemia Salina* nauplii) swimming.	Raphaël Candelier	15	Yes	No
BAC_001	No	74	907x866	*Variable*	*Bacillus Subtilis*	Growing colony of *Bacillus Subtilis.*	Lydia Robert	*Unpublished*	Yes	Yes
BLS_001	No	2,213	1024x1024	7	Black Scavengers	Black scavengers walking in an arena.	Juan Pablo Busso	*Unpublished*	No	No
DRO_001	Yes	1,803	640x360	3	Adult fruit flies	Blow-up of three fruit flies (*D. Melanogaster*) in an Y-maze assay.	Benjamin de Bivort	*Unpublished*	No	No
DRO_002	No	1,589	640x480	91	Adult fruit flies	Fruit flies (D. Melanogaster) walking in an Y-maze assay. Very low resolution.	Benjamin de Bivort	21	No	Yes
DRO_003	Yes	927	1280x720	12	Adult fruit flies	Fruit flies (*D. Melanogaster*) in a multi-chamber assay.	Benjamin de Bivort	*Unpublished*	No	No
DRO_004	No	3,490	602x228	15	Adult fruit flies	Fruit flies (*D. Melanogaster*) in a multi-tubes odor assay.	Benjamin de Bivort	22	No	No
DRP_001	No	700	1224x476	*Variable*	Droplets	Round droplets evolving in a narrowing microfluidic channel.	Lea-laetitia Pontani	16	No	No
DRP_002	No	600	1224x477	*Variable*	Droplets	Polyhedral droplets evolving in a narrowing microfluidic channel.	Lea-laetitia Pontani	*Unpublished*	No	No
DRP_003	No	835	1223x434	*Variable*	Droplets	Polyhedral droplets evolving in a narrowing microfluidic channel.	Lea-laetitia Pontani	*Unpublished*	Yes	No
DRP_004	No	299	640x360	*Variable*	Droplets	Six-layers pattern of droplets in a diverging/converging microfluidic channel.	Bibin M. Jose, Thomas Cubaud	23	No	Yes
DRP_005	No	660	960x730	*Variable*	Droplets	Edge-fluorescent droplets evolving in a narrowing microfluidic channel.	Lea-laetitia Pontani	*Unpublished*	No	Yes
GRA_001	No	1,001	2040x2040	2043	Hard disks	Vibrated isotropic bidisperse discs.	Olivier Dauchot	*Unpublished*	No	Yes
GRA_002	No	1,009	900x900	*Variable*	Hard disks	Motion of dense bidisperse hard particles close to jamming.	Raphaël Candelier, Olivier Dauchot	24	No	Yes
GRA_003	No	165	1470x1469	*Variable*	Vibrated grains	Motion of a pulled intruder in a dense set of bidisperse particles.	Raphaël Candelier, Olivier Dauchot	24	No	Yes
HXB_001	No	1,001	2040x2048	10	Hexbugs	Tiny robots vibrated in a parabolic arena.	Olivier Dauchot	25	Yes	N/A
IND_001	No	2,255	512x512	*Variable*	Plastic parts	Plastic connectors for microfluidics moving on a conveyor belt. Some are isolated, some are overlapping.	Raphaël Candelier	*Unpublished*	Yes	No
IND_002	No	2,463	512x512	*Variable*	PCB	Small printed circuit boards (PCB) moving on a conveyor belt. Some are isolated, some are overlapping.	Raphaël Candelier	*Unpublished*	Yes	Yes
MED_001	No	2,134	640x480	2	Adult Medakas	Two fish (*Oryzias latipes*) following a moving visual pattern.	Hideaki Takeuchi	26	Yes	No
MIC_001	No	693	854x480	2	Adult mice	Two white mice interacting in a dark arena.	Noldus Information Technology	*Unpublished*	Yes	No
PAR_001	No	1,000	1024x1024	*Variable*	Paramecia	Paramecia swimming in an open field.	Alexis prevost	*Unpublished*	Yes	No
ROT_001	No	1,311	800x800	*Variable*	Rotifers	Rotifers (*Rotifera*) swimming in an open field.	Raphaël Candelier	15	Yes	No
SOC_001	No	600	1920x1080	23	Humans	Aerial view of a soccer game.	*Raphaël Candelier*	*Unpublished*	Yes	Yes
TIS_001	No	241	301x301	*Variable*	Neurons	Developing neurons in the hindbrain of a GCaMP3 3dpf zebrafish larva. Total acquisition time: 30 min.	Raphaël Candelier	*Unpublished*	No	Yes
TIS_002	No	241	301x300	*Variable*	Neurons	Developing neurons in the optic tectum of a GCaMP3 3dpf zebrafish larva. Total acquisition time: 30 min.	Raphaël Candelier	*Unpublished*	No	Yes
TRA_001	Yes	1,171	1280x720	*Variable*	Vehicles	Aerial view of traffic.	No Copyright Footage	*Unpublished*	No	Yes
ULT_001	Yes	383	1280x720	*Variable*	Humans	Aerial oblique view of a sequence of Ultimate (flying disk) game.	Paulin Huger	*Unpublished*	Yes	Yes
ULT_002	Yes	953	1280x720	*Variable*	Humans and disk	Aerial top view of a half-field Ultimate (flying disk) game.	Paulin Huger	*Unpublished*	Yes	Yes
ZFA_001	No	15,000	1920x1080	5	Adult zebrafish	Five adult zebrafish interacting.	Gonzalo G. de Polavieja	13	Yes	No
ZFA_002	No	2,000	1218x482	2	Adult zebrafish	Two long-fin (TL) and normal fin (AB) zebrafish interacting.	Benjamin Gallois	*Unpublished*	Yes	No
ZFA_003	No	2,000	1217x471	3	Adult zebrafish	Three Tupfel long-fin (TL) zebrafish interacting.	Benjamin Gallois	*Unpublished*	Yes	No
ZFA_004	No	2,000	1212x472	4	Adult zebrafish	Four zebrafish (TL, AB) interacting.	Benjamin Gallois	*Unpublished*	Yes	No
ZFJ_001	No	200	524x338	14	Juvenile zebrafish	Three weeks-old zebrafish swimming in a flow.	Benjamin Gallois	*Unpublished*	Yes	No
ZFL_001	No	72,000	736x736	39	Larval zebrafish	Two hours of 6 d.p.f. larvae freely swimming in an enclosed arena, recorded at 10Hz.	Raphaël Candelier	*Unpublished*	Yes	N/A
ZFL_002	No	1,130	721x696	5	Larval zebrafish	High-speed imaging of the response of confined zebrafish larvae to an acoustic pulse. Framerate: 1kHz.	Raphaël Candelier	*Unpublished*	Yes	No
ZFL_003	No	1,870	1000x500	3	Larval zebrafish	Three larval zebrafish attracted by a flow with a dye.	Raphaël Candelier	*Unpublished*	Yes	No
ZFL_004	No	14,830	1024x768	24	Larval zebrafish	Larval zebrafish in a 24-multiwell plate.	Elim Hong, Margherita Zaupa	*Unpublished*	No	Yes
