# Supplementary material for: FastTrack: An open-source software for tracking varying numbers of deformable objects
Source: PLoS Comput Biol. 2021 Feb 11;17(2):e1008697. doi: 10.1371/journal.pcbi.1008697 (PMC7904165; doi:10.1371/journal.pcbi.1008697)
Supplement: S2 Table — List of the parameters that can be set in FastTrack for image processing and automatic tracking, as of version 5.1.7. (PDF) [file pcbi.1008697.s002.pdf]

S2 Table. User-defined parameters (v5.1.7)

Category	Name	CLI option name	Range	Unit	Description
Filesystem	Images path	`--path`	-	-	Path to the folder where the images are stored
	Background path	`--backPath`	-	-	Path to a background image (optional)
	Configuration file	`--cfg`	-	-	Path to a configuration file (optional)
Detection	ROI bottom x	`--xBottom`	0 ; image width-1	pixel	Bottom corner x-coordinate of the region of interest. Set to 0 to avoid cropping the image.
	ROI bottom y	`--yBottom`	0 ; image height-1	pixel	Bottom corner y-coordinate of the region of interest. Set to 0 to avoid cropping the image.
	ROI top x	`--xTop`	0 ; ROI xBottom-1	pixel	Top corner x-coordinate of the region of interest. Set to 0 to avoid cropping the image.
	ROI top y	`--yTop`	0 ; ROI yBottom-1	pixel	Top corner y-coordinate of the region of interest. Set to 0 to avoid cropping the image.
	Registration method	`--reg`	0 ; 3	-	Registration method, 0: None, 1: Simple, 2: ECC, 3: Features
	Registration method for background	`--regBack`	0 ; 3	-	Registration method to compute the background. 0: None, 1: Simple, 2: ECC, 3: Features
	Background method	`--methBack`	0 ; 2	-	Method to compute the background. 0: min, 1: max, 2: average
	Number of images for background	`--nBack`	1 ; n_{images}	frame	Number of images to compute the background
	Morphological operation	`--morph`	0 ; 8	-	Morphological operation, 0: None, 1: Erode, 2: Dilate, 3: Open, 4: Close, 5: Gradient, 6: TopHat, 7: BlackHat, 8: HitMiss
	Kernel size	`--morphSize`	1 ; image size	pixel	Size of the kernel used in the morphological operation. It can be omited if no operation is performed
	Kernel type	`--morphType`	0 ; 2	-	Type of the kernel used in the morphological operation, can be omited if no operation are performed, 0: Rect, 1: Cross, 2: Ellipse
	Threshold	`--thresh`	1 ; Inf	intensity	Threshold for the binarization. If Light background is set to 0 (resp. 1), pixels with values less (resp. more) than Threshold will be detected as belonging to an object
	Light background	`--lightBack`	0 ; 1	-	Boolean value indicatting if the background is lighter or darker than the objects to track. 0: background is lighter, 1: background is darker
	Minimum area	`--minArea`	1 ; Image area	pixel	Minimal area of the objects to track
	Maximum area	`--maxArea`	1 ; Image area	pixel	Maximal area of the objects to track
Tracking	Object part	`--spot`	0 ; 2	-	Part of the object used for the matching. 0: head, 1: tail, 2: whole object
	Distance normalization	`--normDist`	0^+ ; ∞	pixel	Normalization factor for distances
	Angular normalization	`--normAngle`	0^+ ; ∞	degrees	Normalization factor for orientation increments
	Area normalization	`--normArea`	0^+ ; ∞	pixel	Normalization factor for area increments
	Perimeter normalization	`--normPerim`	0^+ ; ∞	pixel	Normalization factor for perimeter increments
	Maximal distance	`--maxDist`	1; image size	pixel	Maximal distance for the matching. If an object travels more than this distance, it is not tracked and a new object is created
	Maximal disappearance time	`--maxTime`	1; n_{images}	frame	Maximal time for the tracking. If an object disappears longer than this time, it is not tracked and a new object is created
