# Supplementary figures and images for: FastTrack: An open-source software for tracking varying numbers of deformable objects
Source: PLoS Comput Biol. 2021 Feb 11;17(2):e1008697. doi: 10.1371/journal.pcbi.1008697 (PMC7904165; doi:10.1371/journal.pcbi.1008697)

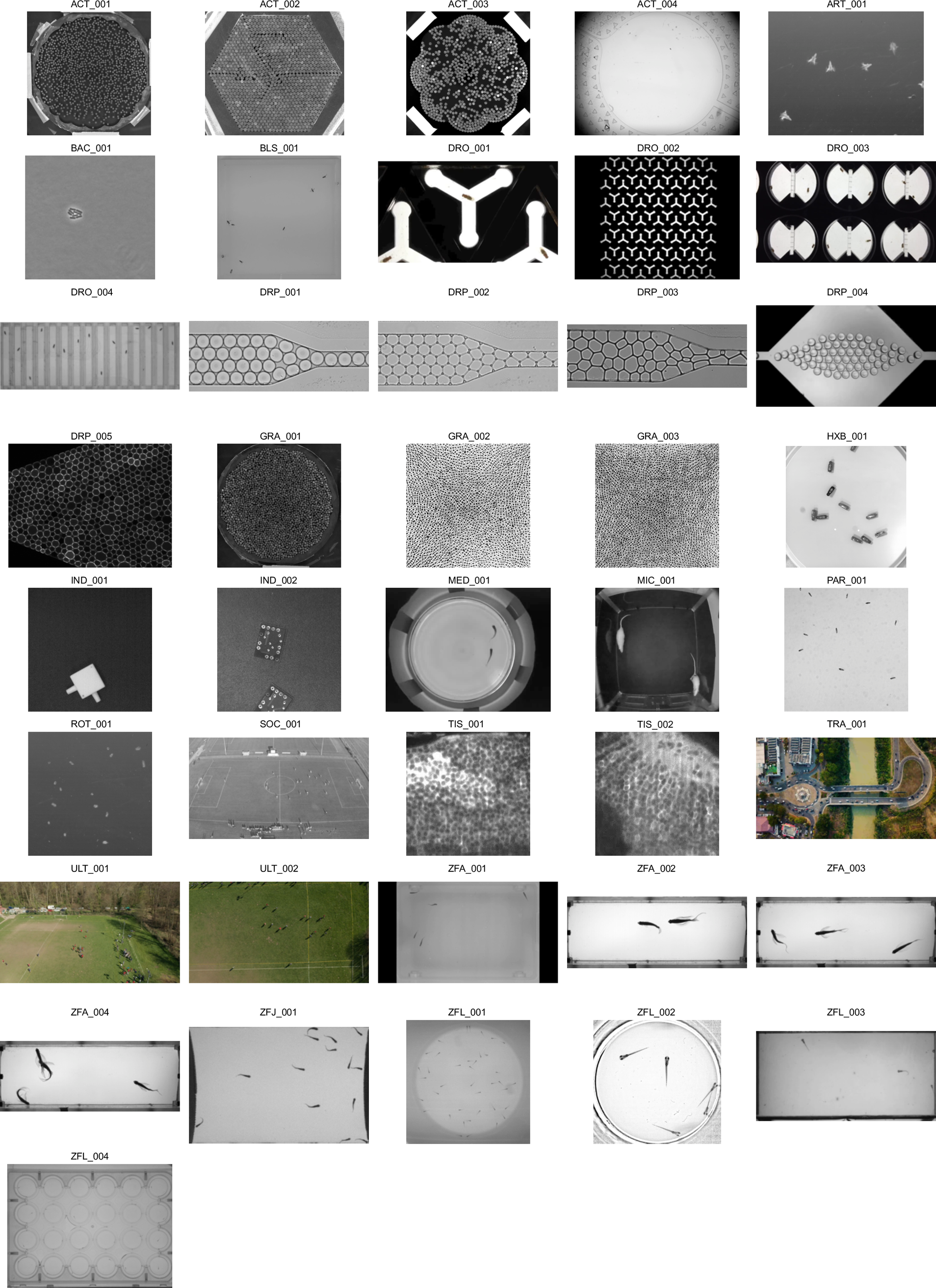

Supplement: S1 Fig — (TIF) [file pcbi.1008697.s003.tif]

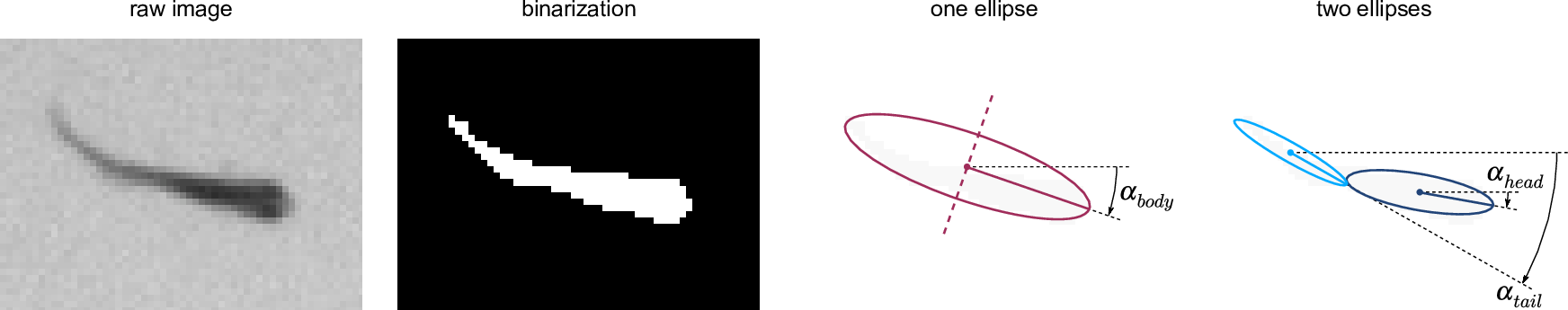

Supplement: S2 Fig — The raw image is binarized and the binary shape is described by one or two ellipses. The position (center of mass) and direction (see text) of the chosen ellipse are used as an input for the matching phase. (TIF) [file pcbi.1008697.s004.tif]

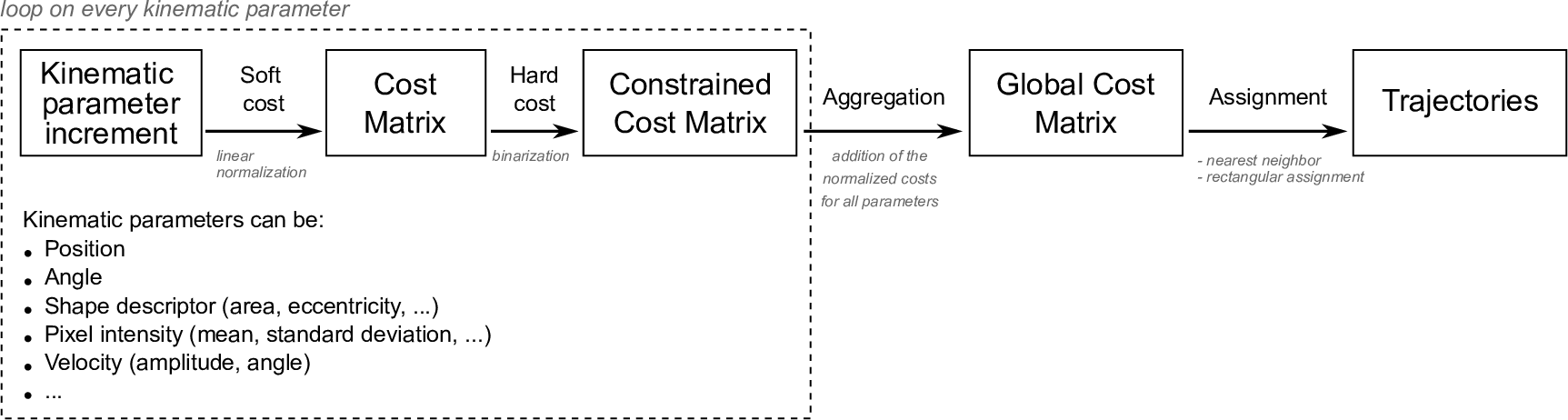

Supplement: S3 Fig — Depending on the system and recording conditions, many kinematic parameters can be employed to define the cost matrix. For each parameter a soft (normalized) and a hard (binarized) terms can be combined and summed to form the General Cost Matrix. An assignment algorithm is then used to produce the trajectories. (TIF) [file pcbi.1008697.s005.tif]

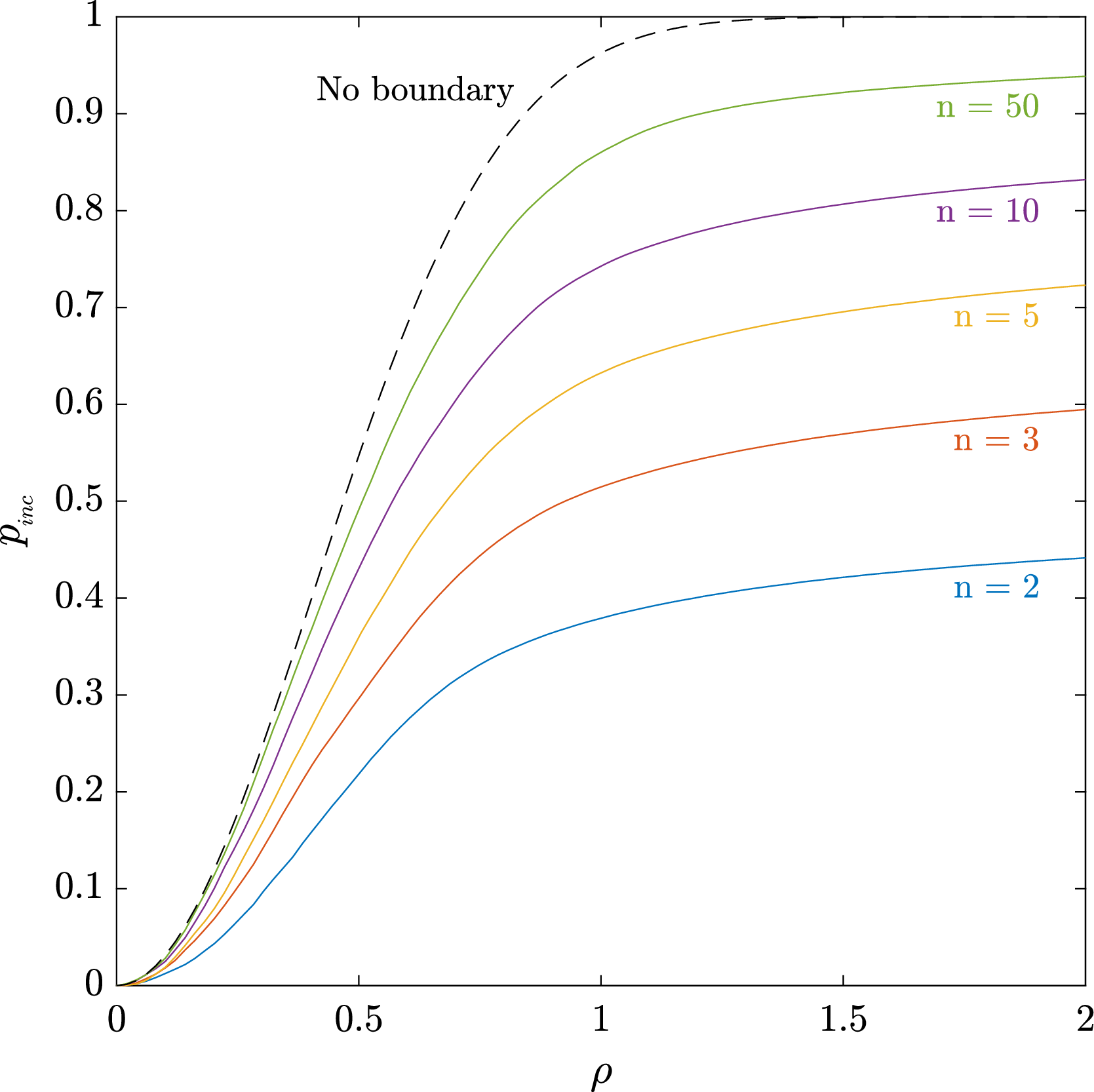

Supplement: S4 Fig — The geometric probability of incursion pinc is computed for a system composed of n punctual objects uniformly distributed at random in a square of size 1, as a function of the reduced displacement ρ for various values of n (plain). pinc(ρ) is also shown for a system without walls (dashed); in this case the curve is independent of the density d. (TIF) [file pcbi.1008697.s006.tif]

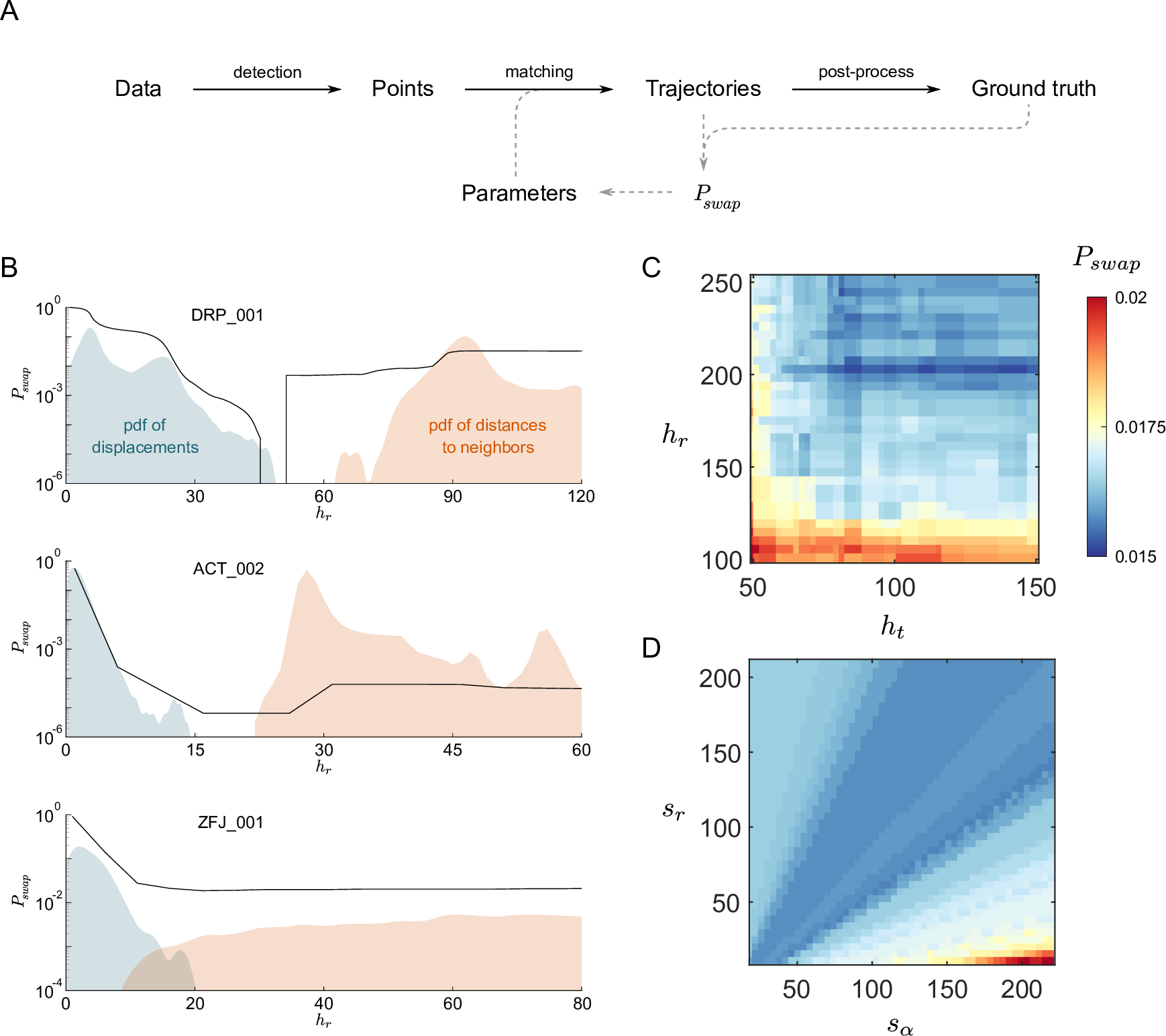

Supplement: S5 Fig — (A) Scheme of the optimization workflow: on top of the detection/matching/post-process flow chart, the ground truth is used to compute Pswap and create a feedback loop on the tracking parameters. (B) Pswap (black) as a function of the maximal distance parameter hr (in pixels) for three typical recordings. Vertical lines for DRP_001 indicate that Pswap drops to 0. The distributions of displacements between successive frames (blue) and of distances to the neighbors (orange) are also shown for comparison. (C) Pswap as a function of the maximal distance parameter hr (in pixels) and the maximal disappearance time ht (in frames) for PAR_001. Soft parameters are set to sr = 95 and sα = 60. (D) Pswap as a function of the normalization distance parameter sr (in pixels) and the normalization angle sα (in degrees) for PAR_001. Hard parameters are set to hr = 210 and ht = 90. (TIF) [file pcbi.1008697.s007.tif]
