# Supplementary material for: FastTrack: An open-source software for tracking varying numbers of deformable objects
Source: PLoS Comput Biol. 2021 Feb 11;17(2):e1008697. doi: 10.1371/journal.pcbi.1008697 (PMC7904165; doi:10.1371/journal.pcbi.1008697)
Supplement: Attachment — Submitted filename: Response_reviewers.pdf [file pcbi.1008697.s014.pdf]

To the editor and the reviewers,

Please find attached a substantially revised version of our manuscript entitled “FastTrack: an open-source software for tracking varying numbers of deformable objects”.

We would first like to thank the reviewers for the quality of their reviews and the many interesting points they raised. Their numerous comments have triggered a much more in-depth analysis of our statistical approach, and the proposition to perform a benchmark strongly strengthened the manuscript. We are now pleased to present a much enhanced version.

In short, we have :

- **improved the software with new features** (more kinematic parameters, computation of P_{inc}, automatic determination of nearly-optimal soft parameters) and bug corrections.
- **completely rewritten the *Results* section**. We moved one figure to supplementary materials (old figure 4), removed two figures (old figures 3 and 5) and added two completely new figures (new figures 3 and 4). We have clarified and strengthened the discussion around the probability of incursion, with comprehensive explanations of how it is calculated and how it can be useful in practice. In particular, we realized that the scaling on P_{swap} that was reported in the first manuscript is much more robust with P_{inc}, so we have moved the whole discussion on this scaling to the probability of incursions. We have also added a detailed description of the new algorithm (already implemented in the latest versions of the software) to determine automatically the soft parameters - those that are usually difficult to set for the end users.
- **added a benchmark** (new figure 5) with two state-of-the-art tracking softwares that demonstrates the speed and high accuracy of FastTrack, even without manual post-processing.

We would also like to report that the FastTrack software has started to spread rapidly among several scientific communities. Since January 2020, the software has been run on 959 unique IP addresses (only for users that have authorized the collection of this information), and we received many bug reports and suggestions for improvement, suggesting that many research groups worldwide actually use it. We believe that publication in PLOS Computational Biology will give it even more visibility and allow many end users to grasp the many computational details of the software.

The authors,

Benjamin Gallois & Raphaël Candelier

Responses to the reviewers

Given the numerous and diverse comments and issues raised by the reviewers, and the fact that some comments actually overlap, we have chosen to write a single answer letter by grouping comments. We then answer the minor points as a list of questions and answers.

Matching algorithm

Reviewer 2
1- hard cost. the authors define h_ij as a hard cost, but in my opinion h_ij is instead a boolean variable associated to the pair i,j: h_ij=1 if r_ij < h_d otherwise it is equal to 0, where r_ij is the distance between i and j, and h_d is a threshold value. Therefore h_ij is a sort of participation variable that defines which pairs have a finite cost.

This is indeed what the hard parameter does and stands for. We have modified the text to clarify this, essentially in the *Matching* subsection. We removed all occurrences of "hard cost" in the manuscript, as it indeed does not exist *per se*.

Reviewer 2
2- soft cost. It is not clear to me how s_d and s_a are chosen. The authors state that these 2 factors are the typical change in position and direction of motion respectively, but they do not give details on how these two factors are computed from the data, and my impression is that s_d and s_a are normalization factors that can be chosen from the users to increase the contribution of one term or the other in the soft cost definition.

Indeed, the soft parameters are normalization coefficients. It may be easier to understand with the revised formula of the cost function (eq. 1, revised version).

The reviewer is also right in saying that it is generally difficult for the end user to determine what those soft parameters should be. As it is much harder to grasp than the hard parameters, several users of the software have asked us about tips on how to do this other by trial and errors. We have thus introduced in the recent versions of the software an algorithm to set these parameters automatically, and we show in the subsection *Automatic tracking parameters* of the new manuscript that it performs quite well since it gives optimal or nearly-optimal tracking parameters. As explained in the new manuscript, it is to be considered as a basis from which the end user can fine-tune the weights given to the kinematic parameters, depending on the specificities of each movie.

Reviewer 2
4- objects disappeareance. The authors should give more details on how the global cost matrix between not-matching objects is performed.
Reviewer 3
While reading the paper, it was not clear to me how the software handles assignment of newly appeared objects, or how intermittently appearing objects are handled.

This is now better explained in the *Matching* subsection of the manuscript.

Probability of incursions and comparison with Pswap

Reviewer 1

In section at line 229 to 264, the authors described the principles and mathematical equations to calculate the number of incursions ninc. Since ninc is important to estimate the post-process load, does the software FastTrack provide an option to calculate this value?

The software now has an option to calculate P_{inc}. It performs a tracking based on the current parameters and outputs the resulting value of P_{inc}. We have not implemented the full timescale analysis presented in the paper (fits of $P_{inc}(\tau)$) in the graphical user interface yet, but for the users interested in this feature it is rather easy to do with the command line interface.

Reviewer 3

- Probability of incursions: The approach is interesting, however explanations are scarce. In particular I could not fully understand Equation 3, with r left undefined.

We have completely rewritten the *Results* section and this part in particular has been beefed up. The distribution $R(\rho)$ is better defined and we give a graphical interpretation of P_{inc} in fig.3B (revised version).

Reviewer 3

The authors show how P_swap can be used to optimize the tracking parameters. This approach is tedious and standard practice. It is interesting to report some methodology, but there is no novelty in this, as far as I can see.

[...]

The general idea that I could grasp is that based on estimated motion (from tracking algorithm) and Voronoi cells, the authors estimate the probability of incursions which may lead to tracking issues (erroneous objects assignments). The authors provide calculations of P_inc they made on several test videos, but they provide no reference to understand how P_inc may be useful.

The general idea of the reviewer is right. We shifted the focus to P_{inc} in the revised version, which is much less tedious to obtain than P_{swap} (as the ground truth is not necessary) and FastTrack can compute it directly. We believe that the new methodology to obtain τ_1 (fig. 3E) has a strong practical interest.

We have kept the discussion on the optimization of P_{swap} but we have moved the corresponding figure in the supplementary materials.

Reviewer 1

In line 258, the authors stated: “...ninc only yields an upper bound of the number of swaps...”

This sentence was unclear and we have removed it in the revised version. Actually, the number of swaps can be higher than the number of incursions in some specific conditions. For instance, we observed chains of swaps even if there is no incursion when the hard threshold is higher than the typical distance between two objects and there is the simultaneous disappearance and appearance of two objects far apart. Though this upper bound makes sense when the hard threshold is correctly set, it cannot be extended to the general case.

Reviewer 1
Plotting the Nswap as a function of ninc for the 41 cases would elaborate this statement.
Reviewer 3
The probability of incursions P_inc may be one of the key contribution of the paper, unfortunately the authors did not connect this value with empirical measurements of P_swap to assess the usefulness of their approach.
[...]
A simple solution would be to manually extract P_swap value of all the videos and compare P_inc to P_swap. This would already help understanding if this estimator is relevant.

We did not connect P_{inc} and P_{swap} in the initial version of the manuscript essentially because the values of P_{swap} heavily depends on the tracking parameters (supp. fig. 5, revised version), and we had no way to determine what tracking parameters should be used. Running brute force optimization on the whole parameter space of every movie would have been prohibitive.

In the revised version, we introduce the automatic determination of the soft parameters and a comparison between the two quantities has been done on this basis (see fig. 4k, revised version).

Datasets and benchmark

Reviewer 2
1- public datasets. The authors state that the algorithm can be applied to a broad class of data. My question is then why they did not test the algorithm on already existing datasets, which is a common practise in the computer vision community.

There are indeed several datasets around, but most of them are focusing on a particular system or on some aspects of the tracking and none is - to our knowledge - really designed for developing general-purpose tracking algorithms.

One of our motivations is that addressing new challenges requires the right tools. We are interested in the development of general-purpose tracking softwares but we couldn't find a database gathering a broad enough collection of systems. We thus initiated our own with the intent of including any type of movie, provided that *i*) it contains something that could be of interest to track and *ii*) that the human eye could track it. There is no other criterion, which we believe is the only honest way to achieve multi-system universality. We estimated that approx. 40 movies was enough to develop our statistical approach and test extensively the possibilities of FastTrack, but the dataset is vowed to grow in the future.

On the other hand, we perfectly understand that exploiting already existing datasets has the major benefit to ease comparisons. In the *Benchmark* section of the revised version, we have tested FastTrack on 5 movies that are provided as examples for idtracker.ai and ToxTrac. We will soon contact the authors of these datasets to see if their movies can be included in the TD² dataset as well.

Reviewer 2
2- tracking data. Many of the data included in the database are interesting from the object detection point of view, but they do not present particular difficulties from the tracking point of view. This is the case of the Drosophila examples in Video1 of the Supplementary Information, where Drosophila move in Y cells that do not communicate, hence tracking is essentially a segmentation

and linking of detected objects within a specific region. This is also the case of the industrial examples of the same Video, where still objects are transported by small platforms.

First, let us note that this is inherent to the design strategy of the dataset (being not selective) and that this does not pose any particular problem. The reviewer is right to say that the compartmented movies are essentially challenging for detection (*e.g.* DRO_002, ZFL_004), but we consider that it is important to show that FastTrack can detect objects with very few pixels, as this is not the case for many softwares including the two ones we used in the *Benchmark* section. Still, all movies in the dataset are useful for varying time-scales analyses: even those that seem really easy to track become challenging at some point when they are undersampled by varying τ (see fig.3, revised manuscript).

Reviewer 1
the advantage of using FastTrack software over others tracking methods is not very clear. The authors mentioned in line 6 that "...there has been countless libraries and softwares..." However, a comparison between the FastTrack software and other tracking methods available in the literature in terms of tracking accuracy and computer cost is missing.
Reviewer 3
The authors claim that their software is fast, with no data to support the claim. Please do a benchmark, with a well characterized hardware, compare to other softwares doing the exact same task.
[...]
There are no tangible results about the tracking performance of the software. I would expect some measurements of speed, of accuracy compared to ground truth, of ergonomics to manually post process results. This could be done using the data set provided. Also, the authors may compare their results to other existing software to better highlight their contribution.

We have compared FastTrack with idtracker.ai and ToxTrac in the *Benchmark* section of the revised version, both in terms of speed and accuracy.

Effectiveness of the software, human labor load and occlusions

Reviewer 1
My second concern is the tracking accuracy and the required human labor for cases with large number of incursions (ninc>1). According to Figure 3D and 4B, for case 'ZFJ_001' with ninc~1, the probability of index swap is Pswap>0.01, which is relatively large. It probably requires a time-consuming manually post-processing procedure to remove the large numbers of index swaps. To broad the applications of the FastTrack, the authors should provide more discussions of the effectiveness of the FastTrack for situations where particle number is high and ninc>1. Is that possible to reduce Pswap, for example, by including more kinematic parameters and optimize them?

ZFJ_001 is a rather short movie so the manual correction of errors is not very long. With the automatic soft parameters (fig. 4E, revised version) we have $P_{swap} \simeq 0.01$ which gives $n_{swap} \simeq 28$. With a little optimisation of the soft parameters we achieved a better tracking requiring only 13 actions during the post-processing to become perfect (see https://www.fasttrack.sh/UserManual/docs/example/ for the step-by-step example in the documentation).

ZFJ_001 is thus indeed a difficult movie to track, but not especially for the incursions. As shown in fig. 3E (revised version), the value of τ_1 for this movie is 1.21 so according to our definition it is slightly oversampled and a better framerate wouldn't really help the tracking. The swaps come from the many occlusions and the fact that the animals swim vigorously and change direction very rapidly and faster than the occlusion time itself: the hypothesis that objects keep their kinematic parameters (other than position) almost constant during occlusions is not verified and the algorithm fails to resolve many occlusions.

In recent versions of the software we added the area and perimeter as kinematic parameters. It did not change the amount of swaps in the particular case of ZFJ_001, though, as many animal sizes are too close to make a proper difference.

Reviewer 2
My impression is that FastTrack is a very powerful tool for objects detection, but the tracking approach is weak in automatically solving occlusions, which is the very core of all tracking algorithms.
[…]
3- occlusion. The matching between detected objects is performed using an Hungarian method, which can only produce one-to-one matches, which is clearly not a way to solve occlusions, but even worse it may produce swapping of identities. Video 1 of Supplementary Information clearly shows what happens when an occlusion occurs, as it is evident in the part of the movie with the 2 mice, but also in other parts of the video. While two objects are occluded the detected occluded detected object is not included in any trajectory and the output of the algorithm consists of 4 short trajectories that are matched before and after the occlusion. I guess that the identities are matched via a manual intervention of the user or via the part of the software related to the objects disappearance.

We would first like to make it clear that resolving the positions during the occlusions is out of the scope of this manuscript. To our knowledge, it is still a very hard and essentially open question. We are not aware of any general purpose software that can handle occlusions, and it is worth noting that the two softwares that we used for the benchmark (idtracker.ai and ToxTrac) also circumvent the problem by ignoring the occlusions and merging the pre and post-occlusion trajectories. The standard in the current softwares is that what happens during the occlusions is lost (and then often interpolated or predicted by a Kalman filter for instance). We'd be happy to hear the reviewer if he/she has some reading to recommend that we would not be aware of.

This being said, it has been shown in ref. [38] (revised version) that the combination of hard and soft terms in the cost function is rather efficient to handle occlusions. Quoting this article: "*the fish occlusion is relatively frequent in the two experiments, but the occlusion detection rate in the proposed method always remains above 0.795, which proves that the proposed method can deal with the fish occlusion well.*". The core idea here is that for most occlusions, and even the severe ones with large area overlaps, the animals do not change their kinematic parameters enough to fool the matching. Of course this highly depends on the biological system, but we observe the same effect with FastTrack in many movies of our dataset, and as a rule of thumb we can estimate that 80 to 90% of the occlusions do not trigger a swap. This is also highlighted by the excellent accuracies obtained by FastTrack before any post-processing presented in the new *Benchmark* section. Properly handling the occlusions then requires human intervention to visually check the trajectories and intervene to correct swaps now and then. This is the reason why we have included the manual post-processing panel in the software, but it is much lighter than the reviewer's assertion that all occlusions need manual intervention.

Finally, we agree that the presence of occlusions is a crucial and very general issue. This is the reason why two movies of the dataset (HXB_001 and ZFL_001) could not be tracked by FastTrack, nor by any other software that we are aware of. As stated in the *Future direction* of the manuscript we are currently working on a general algorithm to resolve occlusions, but this is a totally different work.

Reviewer 2
The algorithm is heavily based on manual checks through post-processing procedure, which is another weakness of the algorithm strictly related to the matching method followed by the authors.
[...]
The entire software is based on post-processing manual check of the algorithm output which is also used to train the matching procedure on data of the same type. My concern about this highly manual component of the algorithm is that this is applicable only on low density datasets of a small number of individuals.

We disagree with these statements. The manual post-processing is applicable to low numbers of errors, which is different from a low density or a low number of objects. There are several movies in the TD² dataset that have more than a thousand objects densely packed and for which FastTrack is performing extremely well. For instance, with an optimized set of parameters for ACT_002 (1085 objects, 1001 frames) we obtained a perfect accuracy (fig.5c, revised version) before any manual post-processing step.

Perhaps the following sentence in the initial version has been misunderstood: "*For the end user, this displaces the workload from the development of complex detection algorithms to the manual post-processing of errors*." We have modified this paragraph in the revised version to make clear that this only concerns the workload associated with the fine-tuning aimed at reaching perfect trackings.

Minor comments

Reviewer 1
Future direction: Considering that objects move at the different speeds, one future direction is to use various hd for different object based on the velocity and acceleration of individual objects calculated from the past trajectories.

We thank the reviewer for this advice and we have included it in the *Future directions* section.

Reviewer 1
In line 227, the authors stated a close to 100% correct tracking. I suggest the author to provide a more quantitatively measure of the tracking accuracy (for example, the magnitude of Pswap) before and after the manual post-processing for the 41 cases.

Unfortunately, this is not possible since we used these trackings as the ground truth, notably in order to calculate P_{swap}. So the calculated P_{swap} would always be zero after manual post-processing. We have modified this sentence to make it clearer that we reached trackings that are only perfect up to a few possible human errors.

Reviewer 1
In line 268, the authors said: "...In order to count the swap..." Does that mean the users have to check the tracking results frame by frame and manually count the number of swaps?

Counting the swaps is done computationally by comparison with the ground truth trajectories, with the formula in eq. 8 (new manuscript). The difficult part is thus to obtain the ground truth trajectories, which indeed requires a manual correction of all remaining errors. This is one of the reasons that make us consider that P_{inc} is a much more useful quantity in practice.

Reviewer 1
Typo in line 141: "interval [0; pi[" and "[0; 2pi[" should be corrected to [0; pi], and [0; 2pi].

This is actually not a typo. We use one of the two notations described in the international standard ISO 31-11 to indicate that one of the endpoints is to be excluded from the set (comprehensive detail can be found on Wikipedia). In these precise cases the values π and 2π are not allowed and replaced by 0 in the software, which is strictly equivalent from a geometrical point of view.

Reviewer 1
In Figure 3C, the meaning of red vertical bars under label "pdf(rho)" is missing.

This figure has been removed in the new version.

Reviewer 2
Fig.2c: how is the computational time normalized?

The time was normalized by the number of pixels in the whole movie using the formula $width * height * n_{frame}$. We clarified this point in the revised version of the manuscript.

Reviewer 2
the authors should give the idea of the method chosen to extract the objects contours

FastTrack uses the built-in function `findContours` from the `OpenCV` library to extract the object's contour, which uses the implementation described in this article:

S. Suzuki, K. Abe, Topological structural analysis of digitized binary images by border following. *Computer Vision, Graphics, and Image Processing*, 30(1):32–46, 1985 algorithm.

This reference was already present in the initial version, we haven't changed it.

Reviewer 2
in Section "Parameters optimization" the authors use the term swap to indicate an error in the matching, but swapping is not the only cause for a tracking error. A second kind of error is a missing match, which happens every time that an object is not included in any match. This last situation is what happens when h_d is too small and there are not possible matching candidates for the objects or for a part of them.

Indeed, errors can be swaps or disappearances and both are taken into account in the computation of P_{swap}, as can be seen in eq. 8 (revised version). In particular, appearances and disappearances are accounted for by the n_{ap} term.

Reviewer 2
developped -> developed

This typo has been corrected.

Reviewer 3
As I tested FastTrack, I had difficulties zooming in particular areas of the image, the authors might want to improve this aspect.

This particular bug was corrected with the commit ed3f9aef01901af93d004c455f428de3024f2f5f and was included in version 5.1.0.

Reviewer 3
Also, the replay section could see some ergonomics improvements. Manual corrections are best carried out in non linear editing mode, with simple controls to go back and forth between frames. I found only one slider to move between frames, and keyboard shortcuts would alternate between translating the image and skipping frames.

The user can move inside a movie in a non-linear mode with the timeline added in the 5.1.0 version. Several keyboard shortcuts are available (from version 4):

- Q/A: go to the previous image.
- D: go to the next image.
- F: delete the selected object on the current image.
- C: enter the number of images where an object has to be deleted.
- G: delete an object from the current image to the current plus the selected number.

Reviewer 3
I also tried to do some manual corrections on a personal video of moving fish, but my attempt was rather unsuccessful. As an example, I faced the case of 3 fish getting close together and the overlay of ids and ellipses made it unclear where each fish was. Moreover, I don't see how to do manual corrections if some objects are not detected during several frames and assignments jump to distant locations (for instance when 3 fish overlap in the video during several frames). It may help to visualize past and future trajectories, and be able to select and drag a detected object to its "real" location, and optionally to recalculate the trajectories of nearby objects in case of a 2-objects swap.

The size and number of ellipses (maximum 2) by objects can be adjusted in the software to improve visibility. Past trajectories can also be displayed to help the user to correct the tracking. In the case of objects overlapping, the maximal area parameter has to be set to avoid false detection, and when the detection is missing in several successive frames the software is usually able to reassign objects identity correctly. In the case of wrong assignments, the user can click on the objects (or select the ids in the toolbox) and invert the ids to correct the wrong assignment by right-clicking (or press the button in the toolbox). After the tracking, all the trajectories and ids are updated and saved.

Reviewer 3
Finally, the software is extracting all the frames of analyzed videos in png format which might take a long time, or be impossible in case of high resolution or long movies (example: 4K 24h @ 1fps could easily yield 260GB of images)

FastTrack is natively able to open video files since version 5.1.0 (commit 70fd1ea26b31e22ecf118b066fe86673481449b0).
