# Supplementary material for: FastTrack: An open-source software for tracking varying numbers of deformable objects
Source: PLoS Comput Biol. 2021 Feb 11;17(2):e1008697. doi: 10.1371/journal.pcbi.1008697 (PMC7904165; doi:10.1371/journal.pcbi.1008697)
Supplement: Attachment — Submitted filename: PCOMPBIO_2020_06_25.docx [file pcbi.1008697.s013.docx]

Review for manuscript: **FastTrack: an open-source software for tracking varying numbers of deformable objects**

This manuscript introduced an open-source software named *FastTrack*, that is generally suitable to track the motion of different types of objects in two-dimensional space. The software incorporated standard imaging processing techniques for object detection, and the Kuhn-Munkres matching algorithms for the temporal tracking. The software also provided a graphical user interface for users to visualize the tracking results, spot tracking errors, and manually correct the errors. The authors also discussed the optimal tracking parameters and frame rate to improve the tracking accuracy. The authors tested the effectiveness of the software on different types of objects covering a wide range of scales. The manuscript is well written, and the workflow of the software is well explained. Developing an open-source software that can be used for general biological and physical systems would be a significant contribution to the scientific communities. I recommend the publication of this manuscript after the authors have considered the following comments and suggestions.

First, the advantage of using *FastTrack* software over others tracking methods is not very clear. The authors mentioned in line 6 that “...there has been countless libraries and softwares...” However, a comparison between the *FastTrack* software and other tracking methods available in the literature in terms of tracking accuracy and computer cost is missing. For cases with *n_inc_*<1, simple tracking algorithm based on nearest neighbor might be sufficient.

My second concern is the tracking accuracy and the required human labor for cases with large number of incursions (*n_inc_*>1). According to Figure 3D and 4B, for case ‘ZFJ_001’ with *n_inc_*~1, the probability of index swap is *P_swap_*>0.01, which is relatively large. It probably requires a time-consuming manually post-processing procedure to remove the large numbers of index swaps. To broad the applications of the *FastTrack*, the authors should provide more discussions of the effectiveness of the *FastTrack* for situations where particle number is high and *n_inc_*>1. Is that possible to reduce *P_swap_*, for example, by including more kinematic parameters and optimize them?

**Minor suggestions:**

In line 227, the authors stated a close to 100% correct tracking. I suggest the author to provide a more quantitatively measure of the tracking accuracy (for example, the magnitude of *P_swap_*) before and after the manual post-processing for the 41 cases.

In section at line 229 to 264, the authors described the principles and mathematical equations to calculate the number of incursions *n_inc_*. Since *n_inc_* is important to estimate the post-process load, does the software *FastTrack* provide an option to calculate this value?

In line 258, the authors stated: “...*n_inc_* only yields an upper bound of the number of swaps...” Plotting the *N_swap_* as a function of *n_inc_* for the 41 cases would elaborate this statement.

In line 268, the authors said: “...In order to count the swap...” Does that mean the users have to check the tracking results frame by frame and manually count the number of swaps?

Typo in line 141: “interval [0; pi[“ and “[0; 2pi[” should be corrected to [0; pi], and [0; 2pi].

In Figure 3C, the meaning of red vertical bars under label “pdf(rho)” is missing.

Future direction: Considering that objects move at the different speeds, one future direction is to use various *h_d_* for different object based on the velocity and acceleration of individual objects calculated from the past trajectories.
